# Intrahepatic Lipid Content and Insulin Resistance Are More Strongly Associated with Impaired NEFA Suppression after Oral Glucose Loading Than with Fasting NEFA Levels in Healthy Older Individuals

**DOI:** 10.1155/2013/870487

**Published:** 2013-05-02

**Authors:** Francis M. Finucane, Stephen J. Sharp, Mensud Hatunic, Alison Sleigh, Ema De Lucia Rolfe, Avan Aihie Sayer, Cyrus Cooper, Simon J. Griffin, David B. Savage, Nicholas J. Wareham

**Affiliations:** ^1^MRC Epidemiology Unit, Institute of Metabolic Science, Addenbrooke's Hospital, P.O. Box 285 Hills Road, Cambridge CB20QQ, UK; ^2^Galway Diabetes Research Centre, School of Medicine, Clinical Science Institute, NUI Galway, Galway, Ireland; ^3^Metabolic Research Laboratories, Institute of Metabolic Science, Cambridge CB20QQ, UK; ^4^Wolfson Brain Imaging Centre, University of Cambridge, Cambridge CB20QQ, UK; ^5^MRC Lifecourse Epidemiology Unit, University of Southampton, Southampton SO166YD, UK

## Abstract

*Introduction*. The mechanisms underlying the association between insulin resistance and intrahepatic lipid (IHL) accumulation are not completely understood. We sought to determine whether this association was explained by differences in fasting non-esterified fatty acid (NEFA) levels and/or NEFA suppression after oral glucose loading. *Materials and Methods*. We performed a cross-sectional analysis of 70 healthy participants in the Hertfordshire Physical Activity Trial (39 males, age 71.3 ± 2.4 years) who underwent oral glucose tolerance testing with glucose, insulin, and NEFA levels measured over two hours. IHL was quantified with magnetic resonance spectroscopy. Insulin sensitivity was measured with the oral glucose insulin sensitivity (OGIS) model, the leptin: adiponectin ratio (LAR), and the homeostasis model assessment (HOMA). *Results*. Measures of insulin sensitivity were not associated with fasting NEFA levels, but OGIS was strongly associated with NEFA suppression at 30 minutes and strongly inversely associated with IHL. Moreover, LAR was strongly inversely associated with NEFA suppression and strongly associated with IHL. This latter association (beta = 1.11 [1.01, 1.21], *P* = 0.026) was explained by reduced NEFA suppression (*P* = 0.24 after adjustment). *Conclusions*. Impaired postprandial NEFA suppression, but not fasting NEFA, contributes to the strong and well-established association between whole body insulin resistance and liver fat accumulation.

## 1. Introduction

Excess intrahepatic lipid (IHL) accumulation (nonalcoholic fatty liver disease, NAFLD) is an important component of the spectrum of metabolic derangements associated with central obesity and insulin resistance [1]. Studies elucidating the mechanistic basis for these associations have suggested that increased circulating nonesterified fatty acids (NEFAs) lead to elevations in IHL [2] and that these NEFAs are generated predominantly from lipolysis in white adipose tissue [3]. Inhibition of glucose oxidation by fatty acids is known to be an important feature of the glucose-fatty acid (or Randle) cycle [4], such that increases in plasma NEFA levels during starvation or in the diabetic state result in greater fat oxidation at the expense of glucose oxidation. Although insulin-mediated suppression of circulating NEFA levels is a robust marker of adipocyte insulin sensitivity [5, 6], elevated fasting NEFA levels are generally considered to be the cause of hepatic fat accumulation in obese insulin-resistant states [7]. Furthermore, a recent study of 42 nonobese adults which measured postabsorptive fatty acid disposal with labeled palmitate found no association between this and whole body or peripheral insulin sensitivity [8]. A formal comparison of the strengths of the associations between fasting and suppressed NEFA levels is therefore warranted. Our first objective was to compare the strengths of the associations, if any, between whole body insulin sensitivity (as the exposure) and fasting NEFA levels versus NEFA suppression after oral glucose loading (at 30 and 60 minutes, as the “outcomes”). For this, we conducted a post hoc, cross-sectional analysis of metabolic and anthropometric data from a cohort of healthy older adults who participated in the Hertfordshire Physical Activity Trial (HPAT) [9].

NEFA suppression is impaired in type 2 diabetes [10] and NAFLD [11], but whether the well-established associations between insulin resistance, impaired NEFA suppression, and IHL persist in apparently healthy older individuals without prevalent NAFLD or diabetes is not known. Also, the extent to which impaired NEFA suppression modulates the accumulation of IHL has not previously been determined. Chronic inflammation within adipose tissue has been implicated in the pathogenesis of peripheral insulin resistance [12], and levels of fat-derived hormones (specifically the leptin : adiponectin ratio, LAR) have recently been shown to correlate well with clamp measures of whole body insulin sensitivity [13]. Therefore, our second objective was to estimate the associations between model-based measures of insulin resistance and impaired NEFA suppression as exposures and IHL as the outcome and to determine the extent to which the associations between insulin resistance and IHL were explained by variations in NEFA suppression.

## 2. Methods

The rationale and design for the Hertfordshire Physical Activity Trial (ISRCTN 60986572) have been described previously [9]. Data reported here relate to post hoc, cross-sectional analyses of volunteers' anthropometric and metabolic characteristics at the time of their entry into the study. Each participant provided written informed consent. The original study protocol was approved by the Hertfordshire Research Ethics Committee (LREC ref. 05/Q0201/23).

Trial participants were recruited from the Hertfordshire Cohort Study, consisting of men and women born in Hertfordshire, UK, between 1931 and 39 and still residing there [14]. Specifically, those who were deemed to be potentially suitable by their general practitioner for inclusion in a supervised aerobic exercise programme and who lived within ten miles of the exercise facility were invited to participate, as described previously [9]. Those with known diabetes, untreated or unstable ischaemic heart disease, or any medical condition that would preclude participation in an exercise programme were excluded from the trial. However, participants with incident diabetes (diagnosed at the time of entry to this study) were included in these analyses. Recruits attended the clinical research facility after an overnight fast. Of 106 who attended the screening visit, six were deemed to be unsuitable for the study because of poor mobility, preexisting diabetes, symptoms or signs suggestive of untreated ischaemic heart disease, or a combination of these factors were excluded. Of the remaining 100, MR imaging and spectroscopy were not performed on 30 individuals who had claustrophobia, cardiac pacemakers, or metal implants. Thus, 70 participants who enrolled in the study had baseline liver spectroscopy measures performed and constitute the cohort described herein.

All measurements were undertaken by trained staff adhering to standard operating procedures. Weight was measured on a Tanita (Tokyo, Japan) scale and height with a Seca (Hamburg, Germany) wall-mounted stadiometer. Waist circumference was measured using a D-loop nonstretch fibreglass tape measure and defined as the midpoint between the lower costal margin and the level of the superior iliac crests. Blood pressure was measured with an oscillometric device (Omron, Kyoto, Japan) using the right arm, after participants were seated quietly for five minutes. A dual energy X-ray absorptiometry (DEXA) scan (Lunar Prodigy Advance, GE Healthcare, Bedford, UK) was used to measure lean mass and body fat percentage [15]. Magnetic resonance measures of intrahepatic lipid (IHL) and visceral adipose tissue (VAT) were conducted on a whole body Siemens 3T Tim Trio scanner (Erlangen, Germany), as described previously [9]. A questionnaire was used to quantify alcohol consumption in units per week.

A standard 75 g oral glucose tolerance test (OGTT) was performed. Fasting samples were taken for glucose, insulin, C-peptide, lipid profile, NEFA, leptin, and adiponectin. Glucose was measured using a hexokinase assay (Siemens, Frimley, UK). Insulin and C-peptide were measured using a fluorometric autoDELFIA immunoassay (PerkinElmer Life Sciences, Turku, Finland). NEFA levels were measured on a colorimetric assay (Roche Diagnostics, Burgess Hill, UK). Leptin and adiponectin were both measured using a DELFIA assay (R&D Systems Europe, Abingdon, UK). After ingestion of glucose, further samples were taken every 30 minutes over two hours. Samples for glucose and lipid profiles were processed immediately, while those for insulin, C-peptide, NEFA, leptin, and adiponectin were spun and frozen for subsequent batch analysis. All samples were processed in the same laboratory. The oral glucose insulin sensitivity (OGIS) model [16] was used to determine peripheral insulin sensitivity based on dynamic insulin and glucose responses during the OGTT, primarily mediated through insulin effects on muscle. Additionally, we used the leptin : adiponectin ratio [13] as an index of whole body insulin sensitivity and the homeostasis model assessment (HOMA) as an index of hepatic insulin sensitivity [17]. The degree of insulin-mediated NEFA suppression was determined by calculating the percentage reduction in NEFA levels 30 and 60 minutes after glucose loading. The area under the concentration curve (AUC) for NEFA during the OGTT was calculated with the trapezium rule.

The anthropometric and metabolic characteristics of the study participants were summarised using means and standard deviations. To estimate the association between each of these characteristics and IHL, linear regression was used with log(IHL) as the outcome, and each characteristic standardised to have mean 0 and variance 1. The models also included age, gender, alcohol consumption (units per week, self-reported), and, where relevant, MRI-derived visceral fat area. For each exposure, the beta coefficient and 95% confidence limits were exponentiated, giving a ratio of geometric mean IHL per standard deviation increase in the exposure. The associations between standardised measures of insulin sensitivity (OGIS, LAR, and HOMA) and IHL were estimated using the same method, with adjustment for age, gender, MRI-derived visceral fat area, and alcohol consumption. The potential confounding effect of NEFA suppression at 30 and 60 minutes was also explored.

## 3. Results

**Figure 1:**
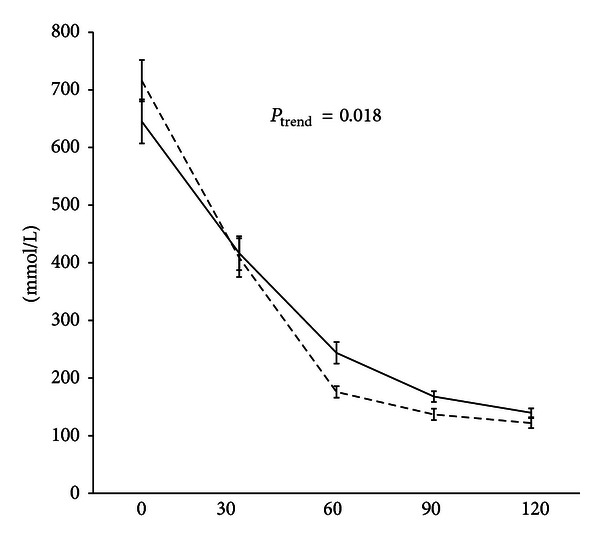
NEFA levels during standard 120-minute 75 g oral glucose tolerance test in participants with intrahepatic lipid ≤5.5% (dashed line) versus those with intrahepatic lipid >5.5%, that is, NAFLD (solid line). Data are presented as mean ± SE. *P*_trend_ values are derived from linear regression modelling with IHL treated as a continuous outcome variable and the exposure being the area under the NEFA curve, adjusted for age, gender, alcohol consumption, and visceral fat area.

Of the 70 HPAT participants included in these analyses, 39 were men. Mean ± SD age was 71.3 ± 2.4 years. Systolic and diastolic blood pressures were 137 ± 18 and 75 ± 9 mmHg, respectively. The median IHL content was 3.6% (range 0.2–34.5%), while 42% of participants had IHL >5.5%, thus exceeding the arbitrary diagnostic threshold for NAFLD [18]. Three participants were found to have incident, asymptomatic type 2 diabetes based on OGTT results and were included in all analyses. Data relating to other anthropometric and metabolic characteristics of the cohort are summarised in Table 1. The associations between these characteristics and IHL are also shown in Table 1. These associations have been standardised in order to allow a comparison of their relative strengths. So, for example, each standard deviation rise in LAR was associated with a 49% increase in IHL, while each standard deviation rise in adiponectin was associated with a 48% reduction in IHL. Different measures of adiposity were positively associated with increased IHL, as expected. There were significant associations between each measure of insulin sensitivity and IHL, in the anticipated directions. NEFA suppression after oral glucose loading was inversely associated with IHL as shown in Table 1 and Figure 1. Results were similar for unadjusted analyses (data not shown).

To assess the relative strengths of the associations between insulin sensitivity and fasting as opposed to suppressed NEFA levels, we standardised OGIS, LAR, and HOMA. There were no significant associations between any indices of insulin sensitivity and fasting NEFA levels (Table 2), nor did HOMA correlate with measures of NEFA suppression. The associations between both OGIS and LAR and NEFA suppression were stronger for the 30-minute than the 60-minute values (Table 2) and were not significant for any of the 120-minute values (data not shown). OGIS and LAR had equivalent (though opposing) strengths of association with NEFA suppression at 30 minutes although the significance of the inverse associations at 60 minutes for LAR was strengthened after adjusting for age, gender, MR VAT, and alcohol consumption.

In order to determine the extent to which impaired NEFA suppression contributes to the observed inverse associations between insulin sensitivity (as exposure) and liver fat (as outcome), we compared these associations before and after adjusting for NEFA suppression (Table 3). After adjusting for NEFA suppression at 30 minutes, the positive association between LAR and IHL was attenuated and lost statistical significance. However, when OGIS or HOMA was treated as the insulin sensitivity measure, there was no attenuation of the association with IHL after adjusting for NEFA suppression. These results were similar after adjusting for NEFA suppression at 60 minutes, as shown. In order to avoid any confounding effects of medications known to influence lipid metabolism, we conducted subgroup analyses excluding those taking statin medications (*n* = 14, 20%) and those taking beta-blockers (*n* = 12, 17%), but these did not change our findings (data not shown).

## 4. Discussion

We found strong and consistent associations between OGIS and LAR (but not HOMA) and NEFA suppression. The absence of any associations with fasting NEFA levels is consistent with other recent observations [19] and suggests that fasting NEFA levels may be influenced by other factors such as catecholamine or growth hormone levels, whereas postprandial NEFA suppression is predominantly determined by insulin. NEFA levels are very promptly suppressed by insulin, so the observation that NEFA suppression at 30 rather than 60 or 120 minutes is a stronger marker of insulin sensitivity is not unexpected.

The similar strengths of associations for LAR and OGIS with NEFA suppression are potentially interesting. (They occurred in opposite directions because the former measures insulin resistance while the latter measures insulin sensitivity.) Given that NEFA suppression is mediated by adipose tissue insulin sensitivity [6], we anticipated that LAR might be more strongly associated with it than OGIS, the latter reflecting insulin-mediated glucose disposal primarily in skeletal muscle, but this was not the case. However, LAR has only previously been shown to correlate with whole body insulin sensitivity rather than fat tissue sensitivity specifically [13], and while leptin and adiponectin are derived exclusively from adipocytes rather than merely acting as markers of adipocyte inflammation, they may actively modulate insulin action in other tissues. The absence of an association between impaired NEFA suppression and HOMA (which is generally regarded as an index of hepatic insulin resistance) suggests that even though the liver is capable of disposal of free fatty acids [20], variations in hepatic insulin sensitivity have a relatively small impact on wholebody fatty acid disposal. Lastly, the finding that the inverse association between NEFA_AUC_ and IHL was twice as strong as that for NEFA suppression at 30 or 60 minutes probably reflects the bidimensional nature of the AUC measure (see Figure 1), where fasting as well as postprandial NEFA levels have a multiplicative effect on this variable.

Our data indicate that excess body fat (particularly visceral fat), insulin resistance, and impaired suppression of NEFA levels after oral glucose loading are all directly associated with increased IHL in older individuals. In order to explore the mechanistic basis for the association between peripheral insulin resistance and NAFLD, we adjusted for NEFA suppression and found that this association was significantly attenuated for LAR but not for OGIS or HOMA. For these analyses, we also adjusted for central adiposity and chose the MRI-derived cross-sectional visceral fat area over other measures of fatness (BMI, body fat %, and waist circumference) because it was most strongly associated with liver fat content (Table 1). However, results were similar when these other measures of fatness were used (data not shown). So reduced suppression of fatty acids explains the association between LAR, but not OGIS or HOMA, and liver fat content. This suggests that while OGIS and LAR are both indices of whole body insulin sensitivity, adipocyte insulin resistance is reflected to a greater extent with LAR than with OGIS or HOMA. These results also suggest that there are other mechanisms apart from impaired NEFA suppression linking insulin resistance and NAFLD, such as dysregulated de novo lipogenesis and hepatocyte endoplasmic reticulum stress [21].

This study has a number of important strengths. Very detailed anthropometric and metabolic characterisation was conducted in each participant, and results were consistent across different measures of body fatness and insulin sensitivity. MR spectroscopy is the most robust noninvasive method for quantifying IHL. Rather than comparing categories of steatosis, body fatness, or glycaemic status, as many studies do, all our measures are continuous and represent the distributions in a relatively healthy cohort of older white participants. The study also has some limitations. It is a post-hoc analysis of data from a subgroup of individuals who were willing to participate in an exercise trial. All participants in the trial were White. Thus, our results may not be generalisable to all older people or those who would be less amenable to an exercise intervention, for whatever reason. Also, only 70% of people who enrolled in the trial had MR imaging at baseline, while others were unwilling or were too large for the scanner, which may have introduced bias.

Nonetheless, the 42% prevalence of NAFLD was higher than we anticipated, and there was a substantial level of metabolic disturbance in this cohort, particularly in relation to the number of individuals with abnormal glucose metabolism during the OGTT. It is important to note that these abnormalities were only detected through participation in the study and were not diagnosed prior to it (and so were not “prevalent” as such). All of these individuals volunteered to participate in a 12-week exercise intervention. Diabetes was one of several exclusion criteria. Nonetheless, three of the 70 individuals (4.3%) had newly diagnosed type 2 diabetes at entry to the study, while a further 27 (38.6%) had abnormal glucose metabolism. None of these had symptoms of hyperglycaemia, nor were they on treatment for it at the time of testing. Therefore, we felt it is appropriate to include them in the analysis. These participants were “apparently healthy,” and within a cohort of this age, a certain level of undiagnosed metabolic disease, be it diabetes or liver steatosis, is to be expected, so to be able to quantify this so precisely in the paper contributes to the novelty of our findings. So our observation too that impaired NEFA suppression does mediate the association between whole body insulin resistance, measured with LAR and liver steatosis.

## 5. Conclusions

In our experience, there is a widely held perception amongst scientists and clinicians in the field of metabolism that fasting NEFA levels are strongly positively associated with insulin resistance. However, in conducting a formal comparison of the relative strengths of the associations between fasting versus suppressed NEFA levels, we have confirmed that the degree of NEFA suppression is far more strongly associated with indices of insulin resistance, namely, OGIS and LAR, and is thus important from a clinical and pathophysiological point of view. We have been careful to take account of confounding factors such as age [22], sex [8], and other factors likely to influence these associations such as alcohol consumption. We believe that the relatively good health of the participants in this study makes the findings described above, particularly in relation to the high prevalence of NAFLD, even more novel and compelling.

## Figures and Tables

**Table 1:** Anthropometric and metabolic characteristics of study participants and their associations (after standardization) with intrahepatic lipid as the outcome measure.

Variable	Mean ± SD	Range	Standardised beta^a^	95% C.I.	*P *
Weight (Kg)^b^	76.0 ± 13.8	48.6–111.7	2.51	[1.86, 3.38]	<0.001
BMI (kg m^−2^)^b^	26.6 ± 3.5	20.7–37.8	2.03	[1.56, 2.64]	<0.001
Waist (cm)^b^	96.7 ± 11.9	72.3–120.5	2.70	[2.05, 3.55]	<0.001
Total fat (%)^b^	32.8 ± 7.5	15.9–48.2	2.26	[1.59, 3.22]	<0.001
MRI VAT (cm^2^)^b^	127.7 ± 65.9	29.2–285.3	2.64	[2.02, 3.44]	<0.001
Alcohol (units/week)	6.5 ± 9.7	0–42	0.86	[0.67, 1.11]	0.24
ALT (iu/L)	28.0 ± 19.9	11–175	1.28	[1.03, 1.60]	0.029
Fasting NEFA (mmol/L)	684.6 ± 208.9	286–1332	1.01	[0.77, 1.33]	0.94
AUC_NEFA_ (mmol/L min)	35435 ± 10353	19680–66180	1.35	[1.06, 1.74]	0.018
NEFA Suppression at 30 minutes (%)	38.6 ± 19.5	−11.2–81.9	0.75	[0.59, 0.96]	0.020
NEFA Suppression at 60 minutes (%)	67.8 ± 15.2	25.2–89.2	0.75	[0.58, 0.96]	0.023
Leptin (ng/mL)	15.3 ± 13.8	0.1–65.3	1.45	[1.002, 2.09]	0.049
Adiponectin (ug/mL)	7.7 ± 4.9	2.2–24.1	0.52	[0.40, 0.69]	<0.001
LAR (ng/ug)	3.0 ± 3.6	0.01–16.3	1.49	[1.05, 2.10]	0.026
Fasting glucose (mmol/L)	5.0 ± 0.5	4.1–6.4	1.49	[1.19, 1.85]	0.001
2-hour glucose (mmol/L)	7.3 ± 2.0	3.9–12.3	1.44	[1.15, 1.80]	0.002
HbA1c (%)	5.7 ± 0.3	4.9–6.4	1.11	[0.88, 1.39]	0.39
Fasting insulin (pmol/L)	64.7 ± 40.3	18.1–288	1.46	[1.13, 1.90]	0.005
HOMA-IR (%)	1.2 ± 0.7	0.4–5.0	1.50	[1.16, 1.92]	0.002
OGIS (mL min^−1^ m^−2^)	409.3 ± 62.7	223–548	0.56	[0.43, 0.72]	<0.001

Intrahepatic lipid was log transformed in all analyses, and then beta coefficients and confidence intervals were back transformed.

^a^Beta represents the ratio of geometric mean IHL per 1 standard deviation increase in relevant exposure. Therefore, standardized beta values > 1 represent a positive association, and values < 1 represent a negative association. All analyses are adjusted for age, gender, alcohol consumption, and MRI-derived visceral fat area (MRI VAT).

^b^These analyses are not adjusted for MRI VAT.

HOMA: homeostasis model assessment.

LAR: leptin : adiponectin ratio.

NEFA: nonesterified fatty acids.

OGIS: oral glucose insulin sensitivity.

**Table 2:** Associations between standardised measures of insulin sensitivity (as exposures) and measures of NEFA metabolism (as outcomes).

	Fasting NEFA			NEFA suppression at 30 minutes			NEFA suppression at 60 minutes		
	*β*	95% CI	*P *	*β*	95% CI	*P *	*β*	95% CI	*P *
Unadjusted									
zLAR	−17.8	[−75.8, 40.3]	0.54	−7.9	[−13.0, −2.8]	0.003	−6.0	[−10.0, −2.1]	0.003
zOGIS	10.4	[-46.4, 67.2]	0.72	9.1	[4.2, 13.9]	<0.001	5.6	[1.6, 9.5]	0.006
zHOMA	15.6	[36.2, 67.4]	0.55	−3.6	[−8.4, 1.2]	0.14	−1.8	−5.6, 2.1	0.35
Adjusted^a^									
zLAR	−31.6	[−105.3, 42.1]	0.39	−11.9	[−18.7, −5.1]	0.001	−7.3	[−12.6, -2.0]	0.008
zOGIS	−25.0	[−88.3, 38.3]	0.43	11.0	[5.4, 16.7]	<0.001	4.4	[−0.2, 9.1]	0.062
zHOMA	49.4	[−4.5, 103.3]	0.07	−3.6	[−9.1, 1.9]	0.19	0.02	[−4.2, 4.2]	0.99

^a^Adjusted for age, gender, MR VAT, and alcohol consumption.

NEFA: nonesterified fatty acids.

zHOMA: standardised homeostasis model assessment.

zLAR: standardised leptin : adiponectin ratio.

zOGIS: standardised oral glucose insulin sensitivity.

**Table 3:** Associations between measures of insulin sensitivity and intrahepatic lipid before and after adjusting for NEFA suppression at 30 and 60 minutes.

	Model 1^a^			Model 2^b^			Model 3^c^		
	*β*	95% CI	*P *	*β*	95% CI	*P *	*β*	95% CI	*P *
LAR	1.11	[1.01, 1.21]	0.026	1.05	[0.96, 1.16]	0.29	1.06	[0.96, 1.16]	0.24
OGIS	0.99	[0.99, 1.00]	<0.001	0.99	[0.99, 1.00]	<0.001	0.99	[0.99, 0.99]	<0.001
HOMA-IR	1.78	[1.24, 2.55]	0.002	1.69	[1.20, 2.38]	0.003	1.79	[1.28, 2.49]	0.001

Intrahepatic lipid was log transformed in all analyses, and then beta coefficients and confidence intervals were back transformed.

^a^Beta represents the ratio of geometric mean IHL per 1 unit increase in relevant exposure. Association with intrahepatic lipid is adjusted for age, gender, MR VAT, and alcohol consumption.

^b^As per model 1 with additional adjustment for NEFA suppression at 30** **minutes.

^c^As per model 1 with additional adjustment for NEFA suppression at 60** **minutes.

HOMA: homeostasis model assessment.

LAR: leptin : adiponectin ratio.

OGIS: oral glucose insulin sensitivity.

## Abbreviations

DEXA: Dual energy X-ray absorptiometry
HOMA: Homeostasis model assessment
HPAT: Hertfordshire Physical Activity Trial
IHL: Intrahepatic lipid
LAR: Leptin : adiponectin Ratio
NAFLD: Nonalcoholic fatty liver disease
OGIS: Oral glucose insulin sensitivity
VAT: Visceral adipose tissue.
